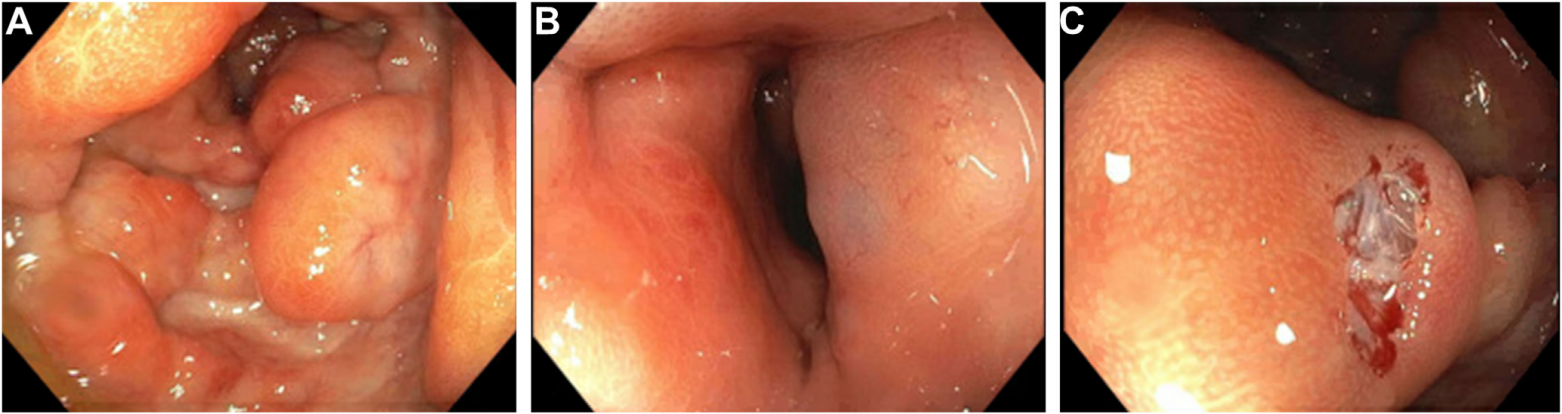# Mycophenolate Mofetil-Induced Pneumatosis Intestinalis

**DOI:** 10.1016/j.gastha.2024.08.003

**Published:** 2024-08-09

**Authors:** Daphne Moutsoglou, Byron P. Vaughn

**Affiliations:** 1 Gastroenterology Department, Minneapolis VA Health Care System and Department of Medicine, University of Minnesota, Minneapolis, Minnesota; 2 Division of Gastroenterology, Hepatology, and Nutrition, Department of Medicine, University of Minnesota, Minneapolis, Minnesota

A 77-year-old male with a history of chronic obstructive pulmonary disease and a liver transplant for hepatitis C virus-induced cirrhosis presented for colonoscopy for diarrhea and hematochezia. He denied fevers, antibiotics, or weight loss. Medications included mycophenolate mofetil (MMF) and prednisone for chronic obstructive pulmonary disease. Outpatient workup showed normal blood work with pending stool studies. Vitals were normal other than baseline hypoxia on room air (89%).

Colonoscopy demonstrated abnormal mucosa from the rectum to the transverse colon (Figure A and B). A single biopsy was taken revealing air-filled cystic lesions (Figure C). No further biopsies were obtained. Computed tomography abdomen pelvis confirmed pneumatosis intestinalis (PI) without any porto-mesenteric gas. Metronidazole was initiated. Pathology revealed MMF colitis. MMF was discontinued, and tacrolimus was initiated. Metronidazole was continued until his computed tomography abdomen pelvis was repeated (one month later), which demonstrated resolution of PI. His diarrhea and hematochezia resolved.

PI is characterized by submucosal/subserosal air-filled cystic lesions. If PI is suspected, avoid biopsy and other endoscopic maneuvers to reduce perforation. Endoscopic findings of PI include pseudolipomas (Figure A), bluish discoloration of mucosa (Figure B), and submucosal air (Figure C). Management is conservative for incidental PI. When symptomatic, treatment includes antibiotics, oxygen therapy, elemental diet, or surgery. Porto-mesenteric gas suggests colonic ischemia with a poor prognosis.

**Figure:**